# Risk of Narcolepsy Associated with Inactivated Adjuvanted (AS03) A/H1N1 (2009) Pandemic Influenza Vaccine in Quebec

**DOI:** 10.1371/journal.pone.0108489

**Published:** 2014-09-29

**Authors:** Jacques Montplaisir, Dominique Petit, Marie-Josée Quinn, Manale Ouakki, Geneviève Deceuninck, Alex Desautels, Emmanuel Mignot, Philippe De Wals

**Affiliations:** 1 Centre for Advanced Research in Sleep Medicine, Hôpital du Sacré-Coeur, and Department of Psychiatry, University of Montreal, Montreal, Canada; 2 Centre for Advanced Research in Sleep Medicine, Hôpital du Sacré-Coeur, Montreal, Canada; 3 Quebec National Public Health Institute (*Institut national de santé publique du Québec*), Quebec City, Canada; 4 Quebec University Hospital Research Centre, Quebec City, Canada; 5 Centre for Advanced Research in Sleep Medicine, Hôpital du Sacré-Coeur, and Department of Medicine, University of Montreal, Montreal, Canada; 6 Stanford Center for Sleep Sciences and Medicine, Stanford University of Medicine, Palo Alto, CA, United States of America; 7 Department of Social and Preventive Medicine, Laval University, Quebec City, Canada; Centers for Disease Control and Prevention, United States of America

## Abstract

**Context:**

An association between an adjuvanted (AS03) A/H1N1 pandemic vaccine and narcolepsy has been reported in Europe.

**Objective:**

To assess narcolepsy risk following administration of a similar vaccine in Quebec.

**Design:**

Retrospective population-based study.

**Setting:**

Neurologists and lung specialists in the province were invited to report narcolepsy cases to a single reference centre.

**Population:**

Patients were interviewed by two sleep experts and standard diagnostic tests were performed. Immunization status was verified in the provincial pandemic influenza vaccination registry.

**Main Outcome Measures:**

Confirmed narcolepsy with or without cataplexy with onset of excessive daytime sleepiness between January 1^st^, 2009, and December 31^st^, 2010. Relative risks (RRs) were calculated using a Poisson model in a cohort analysis, by a self-controlled case series (SCCS) and a case-control method.

**Results:**

A total of 24 cases were included and overall incidence rate was 1.5 per million person-years. A cluster of 7 cases was observed among vaccinated persons in the winter 2009–2010. In the primary cohort analysis, 16-week post-vaccination RR was 4.32 (95% CI: 1.50–11.12). RR was 2.07 (0.70–6.17) in the SCCS, and 1.48 (0.37–7.03) using the case-control method. Estimates were lower when observation was restricted to the period of pandemic influenza circulation, and tended to be higher in persons <20 years old and for cataplexy cases.

**Conclusions:**

Results are compatible with an excess risk of approximately one case per million vaccine doses, mainly in persons less than 20 years of age. However, a confounding effect of the influenza infection cannot be ruled out.

## Introduction

Narcolepsy is characterized by the occurrence of excessive daytime sleepiness (EDS), which is usually the most disabling feature of the disease [1]–[3]. EDS is exacerbated when the patient is physically inactive like reading, watching television or driving a car. The other major manifestation of narcolepsy is cataplexy, consisting of sudden drops of muscle tone triggered by emotions such as laughter, anger or surprise. The onset of the disease is usually during adolescence and young adulthood. The manifestations of the disease can be alleviated by medical treatment and their consequences mitigated through adaptation but there is no cure. Narcolepsy is caused by a selective loss of hypocretin-producing cells in the hypothalamus, presumably the consequence of an autoimmune disorder, with a genetic predisposition [4]–[5]. Environmental factors that could trigger the occurrence of narcolepsy include head trauma, stressful life events or infections [6]–[8].

In 2010–2011, an association between the administration of an inactivated adjuvanted (AS03) A/H1N1 pandemic vaccine produced in Dresden, Germany, (Pandemrix, GlaxoSmithKline Biologicals, Wavre, Belgium) and narcolepsy was reported mostly in children in some European countries [9]–[13]. These cases had common features: severe sleepiness and presence of cataplexy, abrupt onset and rapid evolution, presence of the HLA DBQ1*0602 genetic marker, very low CSF hypocretin-1 levels, and positive Multiple Sleep Latency Test (MSLT) test [14]. At the request of the European Medicine Agency, the vaccine manufacturer funded an independent study in the province of Quebec, Canada, where a similar pandemic vaccine produced in Quebec City, Canada, had been used (Arepanrix, GlaxoSmithKline Biologicals, Wavre, Belgium) and where population-based epidemiologic studies had been conducted to assess the risk of other adverse outcomes [15]–[16]. Such study is important as different adjuvants belonging to the same class are likely to be used for other pandemic influenza vaccines in the future.

## Methods

### Study population and case definition

The study population included all persons 6 months of age or more residing in the province of Quebec (Census data = 7,817,449 as of July 1^st^, 2009). At the end of the mass immunization campaign, 57% of the target population (≥6 months of age) had been immunized, the vast majority (96%) with one dose of the AS03 vaccine [15]. The primary outcome of interest was narcolepsy with or without cataplexy with onset of EDS during the period of January 1^st^, 2009 to December 31, 2010.

### Identification of cases

In Quebec, patients with complex sleep disorders are usually seen by neurologists or lung specialists in private clinics or hospital outpatient services. When a diagnosis of narcolepsy is suspected, patients are referred to sleep laboratories situated in tertiary care hospitals for special investigations and treatment. The main reference centre is situated at the ‘Hôpital du Sacré-Coeur de Montréal’ (HSCM). Between June 2011 and June 2012, all adult and pediatric neurologists and lung specialists in the province were contacted by letter through their association and invited to report any confirmed or suspected narcolepsy case with onset during the study period. Directors of sleep laboratories, key neurologists and lung specialists were also contacted individually and invited to participate. Records of all patients who consulted for EDS at HSCM were also reviewed. A first screening was performed using available medical records. All patients meeting inclusion criteria (high suspicion of narcolepsy and probable date of onset within study period) were formally invited to participate and to be examined at HSCM (if not already done) for in-depth interview and standard diagnostic tests (details are provided in Table S1). Afterwards, information on immunization status was extracted from the provincial pandemic influenza immunization registry which was established at the time of the mass immunization campaign in 2009. For each person immunized, it contains precise information on the date of vaccine administration and the type of vaccine used. Recruitment extended up to June 30, 2012. The research protocol and the consent procedures were approved by the “Comité d’éthique de la recherche de l’Hôpital du Sacré-Coeur de Montréal”. All participants provided a written consent. The consent of a parent and assent of the patient were requested for those less than 18 years of age.

### Classification of cases

All suspected cases were confirmed and classified by two experienced sleep specialists (JM and AD) using all the information available. At this stage, the sleep specialists were blind as to the immunization status of the patient. The criteria proposed by the Brighton Collaboration Narcolepsy Working Group were used to classify narcolepsy cases according to 3 levels of certainty, based on clinical signs and symptoms, hypocretin-1 CSF concentration, and results of daytime MSLT [16]. In cases of discordant opinions, a consensus process was initiated between the two experts.

### Determination of the date of disease onset

For each confirmed case, the determination of the most probable date of disease onset (first occurrence of EDS and/or cataplexy symptoms) was established on the basis of the extensive questionnaire completed at the sleep clinic from the interview of the patient, and for children, from an additional interview with a parent. Most importantly, existing medical files were also requested (with patients’ authorization) and reviewed for a date of onset of first symptoms reported at the first medical visit for excessive somnolence and/or cataplexy. For most patients (n = 15), the date of onset of symptoms was established at the first clinical visit. For these patients, the mean delay between the date of onset and the date of the medical visit was 2.9 months. These dates were also corroborated by the detailed interview at the sleep center. For the rest of patients (n = 9), there was no report of a date of symptoms onset at the time of the first medical visit. In these cases, the assessment of the date of onset was determined through the medical interview at study intake. However, these 9 patients had several first medical visits between onset of symptoms and final assessment date which served as indicators of onset date. The final date could be a precise day or a given (usually short) time period (the median day of the period being used in statistical analyses). Confirmed narcolepsy cases with a date of onset clearly outside the 2009–2010 study period were excluded.

Thereafter, each case was classified according to exposure to the vaccine: (1) not vaccinated, (2) vaccinated after disease onset, (3) vaccinated before disease onset and within a time (risk) period compatible with the development of the disease, (4) vaccinated before disease onset but outside the risk period. The third category represents those exposed and the other categories are considered as not exposed. In the primary analysis, the post-vaccination risk period was defined as 16 weeks (112 days), not including the day of vaccine administration, based on series of cases reported in Canada and in Europe [11], [14]. Sensitivity analyses were also performed using 8 weeks (56 days), 24 weeks (168 days) and one year (365 days) risk periods.

### Statistical analyses

The primary statistical analyses were performed using Brighton level 1 cases (narcolepsy with cataplexy and hypocretin deficiency), level 2 cases (narcolepsy with cataplexy and specific MSLT abnormalities) and level 3 cases (narcolepsy without cataplexy but with specific MSLT abnormalities). Characteristics of narcolepsy cases were described using absolute numbers, range, median, means and proportions. The Fisher exact test was used to compare proportions and the Wilcoxon rank test for comparing medians. Statistical analyses were performed using SAS 9.3 software (SAS Institute, Cary, NC). Statistical significance was established for p value<0.05 (two-sided tests).

First, the narcolepsy incidence rate in the exposed population was calculated using post-vaccination risk periods as defined above (16 weeks in the primary analysis). Incidence rates in the non-exposed population were calculated by combining the experience of persons not vaccinated and the experience of those vaccinated and observed up to the date of vaccine administration and from the end of the post-vaccination risk period to the end of the study period (with no buffer) in the base analysis [17]. Age- and gender-adjusted rate ratios (RRs) and 95% confidence intervals (95% CI) were estimated using a Poisson model. Attributable risks (per million vaccine doses) were calculated as the number of narcolepsy cases per million vaccine doses observed during a specific risk period and multiplied by (RR−1)/RR.

In order to control for seasonality and circulation of the pandemic virus in 2009, other analyses were performed, curtailing the observation to the period May 1^st^, 2009 (beginning of the first pandemic wave) to March 31^st^, 2010 (6 months after the start of the second pandemic wave), and to the period between October 4^th^, 2009 (beginning of the second pandemic wave) and March 31^st^, 2010 (6 months after the start of the second pandemic wave). A final analysis was performed, comparing narcolepsy rates among vaccinated persons only (post-vaccination period v.s. pre-vaccination period), among non-vaccinated persons only (post-vaccination period v.s. pre-vaccination period); among vaccinated and non-vaccinated persons during the pre-vaccination period, and among vaccinated and non-vaccinated persons during the pre-vaccination period.

The self-controlled case series (SCCS) method, as described by Whitaker and colleagues [18] was also used for estimating RRs. In this method, the analysis was restricted to narcolepsy patients who had been vaccinated. In the base model, the observation period extended from 6 months (183 days) prior vaccine administration to 6 months after the date of vaccine administration. The risk period was defined as 16 weeks (112 days) following vaccine administration. To take into account the uncertainty in the definition of the risk period, a buffer period (17–24 weeks post-vaccination) was used. Other pre- and post-vaccination observation periods were used as the reference in sensitivity analyses, including or excluding a buffer period (which was then included in the control period).

Finally, a case-control approach was used [19], which is a variant of the ‘case-coverage’ method’ used in the UK study [11]. In this secondary analysis, only cases with onset after the beginning of the mass immunization campaign (October 24, 2009) were included. Using population and immunization registry data, each narcolepsy case was matched to a large number of controls: all persons of the same gender and of the same single-year age-group in the target population. The exposure was vaccinated or not before the day of disease onset of the case. Odds ratios (ORs), which are approximations of rate ratios, and 95% CI were calculated by conditional logistic regression.

## Results

### Identification of narcolepsy cases

A retrospective review of medical charts of all patients who consulted for somnolence at the sleep clinic of the HSCM between January 1^st^, 2009 and July 21, 2011 was performed. Of these, 588 cases were excluded because they received another diagnosis (e.g. diagnosis of narcolepsy ruled out clinically or based on PSG results) or because onset of first symptoms was prior to January 1^st^, 2009 or later than December 31^st^, 2010. This was determined either by date of referral for somnolence, by the fact that the subject already had a prior visit for the same problem (follow-up visit) or a clear indication by the referring physician that onset of somnolence was outside the target period. Three patients were lost to follow-up and another refused to participate. Out of 33 patients who were retained for a final assessment including standard PSG and MSLT (performed at HSMC in 30 cases), 9 were further excluded as they did not meet Brighton diagnostic criteria. Thus, 24 confirmed narcolepsy cases were retained for statistical analyses (3.8% of the total investigated). Sixteen of these patients had been vaccinated and a precise date of vaccine administration was available for all of them. Details on study patients are presented in Table S2.

### Exposure assessment

For 21 out of 24 patients, the date of onset of first symptoms of narcolepsy could be unambiguously assessed (less than one-month apart between the two sleep specialists). The 15^th^ day of the month was arbitrarily selected when the exact date was missing. For the three other patients, establishing a precise date of disease onset was more problematic. One patient reported somnolence as early as September 2008 but this was mild and symptoms became more severe in August 2009 with the onset of cataplexy. This patient was excluded from the primary analyses and included in sensitivity analyses on cataplexy. This patient was vaccinated in November 2009 and was considered as not exposed in all scenarios. For a second patient not vaccinated, EDS occurred in the period November–December 2009. For a third patient, EDS occurred in the period October–November 2009 (probably in early November) and the date of vaccination was November 15, 2009. Arbitrarily, disease onset was assigned on November 1^st^, 2009 and the case was considered as not exposed in the primary analysis. A sensitivity analysis was performed considering this case as exposed (vaccination before disease onset). The date of vaccination relative to disease onset (before/after) was thus assessed in a non-equivocal way for 15 out of 16 vaccinated patients.

### Characteristics of narcolepsy cases

Sixteen out of 24 patients had developed cataplexy (Table 1). This may change in the future as cataplexy symptoms may appear late in the course of the disease. Five patients were classified as Brighton level 1, 11 as level 2, and 8 as level 3. The mean age of the total sample was 24 years, ranging from 6 to 55 years. Although the difference was not statistically significant, exposed cases tended to be younger than non-exposed cases. The male proportion was 50% in the total sample. The proportion of cases positive for the HLA DQB1*0602 marker was 79% (19/24) and this was similar between exposed and non-exposed cases. Interestingly, the HLA marker was present in all of the 16 cases with cataplexy but only in 3 of the 8 cases without cataplexy. The mean interval between the onset of first symptoms and occurrence of cataplexy was 53 days and tended to be shorter for exposed (24 days) than for non-exposed cases (72 days). The average time interval between the onset of first symptoms and the first medical visit was 149 days, shorter for exposed (66 days) than for non-exposed cases (191 days).

**Table 1:** Distribution of 24 narcolepsy cases according to exposure status and patients’ characteristics.

Patient’s characteristics Mean ± SD/median/range	All cases (n = 24)	Not vaccinated or vaccinated after disease onset (n = 16)	Vaccinated before disease onset (n = 8)	p value*
Age (years)	24.0±14.3	26.1±13.1	19.9±16.6	0.20
	18.1	28.2	13.7	
	6–55	8–55	6–50	
Age group				
<20 years of age	13 (54%)	7 (44%)	6 (75%)	0.21
≥20 years of age	11 (46%)	9 (56%)	2 (25%)	
Male proportion	12 (50%)	10 (62.5%)	2 (25%)	0.19
Cases with cataplexy	16 (67%)	10 (63%)	6 (75%)	0.67
Cases with hypnagogic hallucinations	14/23 (61%)	9 (56%)	5/7 (71%)	0.66
Cases with sleep paralysis	10 (42%)	7 (44%)	3 (38%)	1.00
Cases with all signs and symptoms	5 (21%)	3 (19%)	2 (25%)	1.00
Diagnostic certainty				
Brighton level 1	4 (17%)	2 (12.5%)	2 (25%)	0.73
Brighton level 2	12 (50%)	8 (50%)	4 (50%)	
Brighton level 3	8 (33%)	6 (38%)	2 (25%)	
HLA DQB1*0602 positive	19/24 (79%)	12/16 (75%)	7/8 (88%)	0.63
Interval between onset first sleep	53.3±93.1	72.3±116.9	24.8±27.5	1.00
symptoms and onset of cataplexy (days)	21	0	23	
	0–330	0–330	0–91	
Interval between disease onset and first	149.1±187.8	190.9±218.9	65.5±35.7	0.42
medical visit (days)	70.5	77.5	52.5	
	3–799	3–799	32–124	
Interval between disease onset and	427.8±244.52	482.1±239.3	319.3±231.4	0.15
confirmatory laboratory investigations	414	422	246	
(days)	79–903	170–903	79–756	

*Fisher exact test for proportions and Wilcoxon rank test for medians.

### Distribution of cases over time

**Figure 1:**
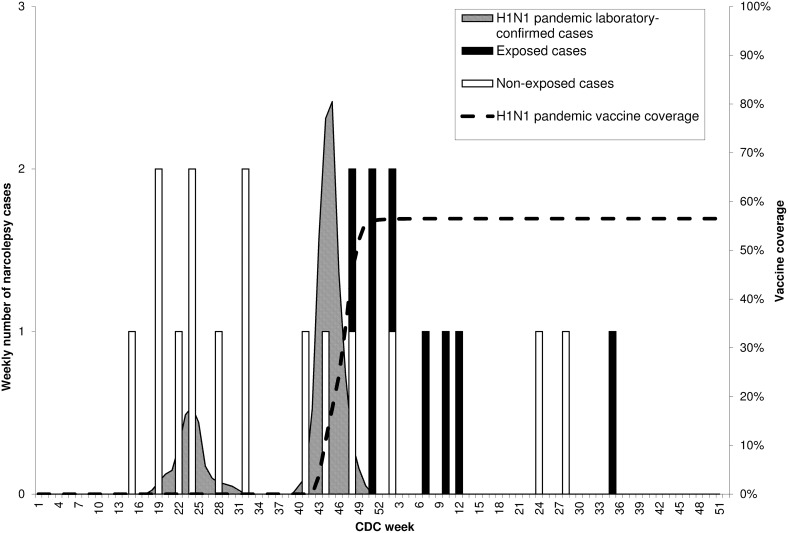
Distribution of dates of onset of 23 narcolepsy cases according to CDC week, in province of the Quebec, 2009–2010.

No case was reported with disease onset before week 15, 2009 or after week 35, 2010 (Figure 1). No seasonal pattern (12-month cycle) was observed, but cases occurred in clusters. A first cluster of narcolepsy cases was observed in the spring-summer of 2009, at the time of the first A/H1N1 influenza pandemic wave. A second cluster was observed in the winter 2009–2010, after the second pandemic wave and the mass immunization campaign. In the second cluster, 7 of the 11 cases were patients who developed symptoms shortly after vaccine administration. These seven cases, considered as exposed, included mainly persons less than 20 years of age (5/7), for whom the onset of narcolepsy was abrupt and cataplexy rapidly developed (6/7).

### Risk estimates using the cohort method

The average incidence rate in the study population including 23 narcolepsy cases with onset in 2009–2010 was 1.5 per million person-years (p-y), 2.0 in 2009 and 1.0 in 2010.

In the base cohort analysis, narcolepsy incidence in vaccinated persons during the 16-week period following vaccine administration was 0.52/100,000 p-y and the rate was 0.11/100,000 p-y in the group not exposed, for an age- and gender-adjusted RR of 4.58 (95% CI: 1.59 to 11.77). The absolute attributable risk estimate was close to 1 case per million vaccine doses (Table 2). When the post-vaccination risk period was extended to 365 days post-vaccination or to the end of the study period (December 31^st^, 2010), risk estimates were lower and no longer significant. On the contrary, when the post-vaccination risk period was shortened (8 weeks), RRs were of the same magnitude than in the base model and statistically significant. When the observation period was restricted to the period of circulation of the pandemic virus (1^st^ and 2^nd^ wave or 2^nd^ wave only), RRs were comprised between 2 and 4 and were not significant.

**Table 2:** Risk of narcolepsy ± narcolepsy associated with A/H1N1 (2009) vaccination using the cohort method according to observation period and post-vaccination risk period, in Quebec, 2009–2010.

Observation period	Risk period from dateof vaccination to:	No cases			Incidence rate			Age- and gender-adjusted risk ratio				Attributable cases/million doses(95% confidence intervals)
		*E+*	*E−*	*Total*	*E+*	*E−*	*Total*	*RR*	*IC_inf_*	*IC_sup_*	*P-value*	
January 1st 2009, toDecember 31, 2010	End study periodDecember 31st, 2010	8	15	23	0.164	0.253	0.148	1.06	39.00	2.68	1.000	0.25 [−2.83 to 1.13]
	One year (365 days)post-vaccination	8	15	23	0.181	0.464	0.148	1.24	0.45	3.13	0.650	0.46 [−2.21 to 1.23]
	24 weeks (168 days)post-vaccination	7	16	23	0.344	1.039	0.148	2.73	0.95	7.04	0.063	1.04 [−0.08 to 1.36]
	16 weeks (112 days)post-vaccination*	7	16	23	0.516	1.237	0.148	4.32	1.50	11.12	0.007	1.24 [0.53 to 1.44]
	8 weeks (56 days)post-vaccination	4	19	23	0.590	0.709	0.148	4.36	1.08	13.15	0.039	0.71 [0.07 to 0.84]
May 1st 2009, toMarch 31 2010	End study periodDecember 31st, 2010	7	12	19	0.448	0.822	0.266	1.91	0.63	5.28	0.272	0.82 [−0.93 to 1.28]
	One year (365 days)post-vaccination	7	12	19	0.448	0.822	0.266	1.91	0.63	5.28	0.272	0.82 [−0.93 to 1.28]
	24 weeks (168 days)post-vaccination	7	12	19	0.448	0.822	0.266	1.91	0.63	5.28	0.272	0.82 [−0.93 to 1.28]
	16 weeks (112 days)post-vaccination	7	12	19	0.516	0.947	0.266	2.33	0.77	6.43	0.138	0.95 [−0.47 to 1.34]
	8 weeks (56 days)post-vaccination	4	15	19	0.590	0.549	0.266	2.39	0.58	7.52	0.233	0.55 [−0.66 to 0.78]
October 4 2009, toMarch 31 2010	End study periodDecember 31st, 2010	7	3	10	0.448	0.133	0.262	2.73	0.62	16.45	0.234	1.11 [−0.97 to 1.49]
	One year (365 days)post-vaccination	7	3	10	0.448	0.133	0.262	2.73	0.62	16.45	0.234	1.11 [−0.97 to 1.49]
	24 weeks (168 days)post-vaccination	7	3	10	0.448	0.133	0.262	2.73	0.62	16.45	0.234	1.11 [−0.97 to 1.49]
	16 weeks (112 days)post-vaccination	7	3	10	0.516	0.122	0.262	3.63	0.82	21.82	0.098	1.21 [−0.35 to 1.51]
	8 weeks (56 days)post-vaccination	4	6	10	0.590	0.191	0.262	2.73	0.57	11.55	0.229	0.61 [−0.68 to 0.83]

*+: Cases with onset after vaccination during risk period; E−: Cases not vaccinated or with onset before vaccination or after end of risk period.*

*Primary analysis defined a priori.

Risk estimates were higher in persons less than 20 years of age (5 cases exposed *v.s.* 7 cases not exposed in base analysis; RR = 6.39; 95% CI: 1.60 to 23.38), than in those 20 years of age or older (2 cases exposed *v.s.* 9 cases not exposed in base analysis; RR = 2.44; 95% CI: 0.26 to 11.80) (details in Tables S3−S4).

When the date of first medical visit was used to define disease onset instead of that of EDS occurrence, the total number of cases retained in the base-case analysis was 20 (3 patients had their first visit in 2011), including 3 exposed and 17 considered as not exposed, for a RR of 1.73 (95% CI: 0.32 TO 5.99) (details of sensitivity analyses are in Tables S5−S9).

### Risk estimates using the self-controlled case-series method

In the base analysis including a post-vaccination buffer period, RR during the 16-week post-vaccination period was 2.07 (95% CI: 0.70 to 6.17). RR was 2.96 (95% CI: 0.71 to 12.39) among persons less than 20 years of age and was 1.18 (95% CI: 0.20 to 7.09) in those older. RRs tended to be higher when the analysis was restricted to Brighton levels 1–2 cases and when the buffer period was excluded (Table 3).

**Table 3:** Risk of narcolepsy ± narcolepsy associated with A/H1N1 (2009) vaccination using the ‘self-controlled case-series method’ according to age and diagnostic classification, in the province of Quebec, 2009–2010.

Observation period	Post-vaccinationrisk period	Post-vaccinationbuffer period	Brighton level	No of casesin analysis	Relative risk	95% confidenceinterval	Attributable risk per million doses (95% confidence intervals)
All ages							
6 months (183 days) beforevaccination to 6 monthsafter vaccination	16 weeks (112 days)	Weeks 17–24(days 113–168)	1–3	13	2.07	0.70 to 6.17	0.82 [−068 to 1.32]
			1–2	9	3.55	0.89 to 14.21	0.97 [−0.17 to 1.26]
		No buffer	1–3	13	2.66	0.89 to 7.90	0.99 [−0.19 to 1.38]
			1–2	9	4.55	1.14 to 18.21	1.06 [0.17 to 1.28]
Persons 6 months to <20 years of age							
6 months (183 days) beforevaccination to 6 monthsafter vaccination	16 weeks (112 days)	Weeks 17–24(days 113–168)	1–3	8	2.96	0.71 to 12.39	2.98 [−1.84 to 4.14]
			1–2	5	7.10	0.79 to 63.59	3.09 [−0.96 to 3.54]
		No buffer	1–3	8	3.79	0.90 to 15.89	3.31 [−0.50 to 4.22]
			1–2	5	9.11	1.02 to 81.48	3.21 [0.07 to 3.56]
Persons ≥20 years of age							
6 months (183 days) beforevaccination to 6 monthsafter vaccination	16 weeks (112 days)	Weeks 17–24(days 113–168)	1–3	5	1.18	1.19 to 7.09	0.09 [−2.58 to 0.52]
			1–2	4	1.78	0.25 to 12.61	0.26 [−1.81 to 0.56]
		No buffer	1–3	5	1.52	0.25 to 9.08	0.21 [−1.81 to 0.54]
			1–2	4	2.27	0.32 to 16.16	0.34 [−1.28 to 0.57]

Using the whole observation period (January 1^st^, 2009 to December 31^st^, 2010) and the 16-week post-vaccination reference period as in the base cohort analysis with no buffer, the all-age RR for Brighton levels 1–3 was 4.82 (95% CI: 1.75 to 13.31).

### Risk estimates using the case-control method

This analysis was restricted to 13 narcolepsy cases with onset on or later than October 24, 2009, the first day of the mass immunization campaign. There were 8 cases with disease onset after vaccine administration and 5 unvaccinated cases, for an OR of 1.48 (95% CI: 0.37 to 7.03). OR was 3.21 (95% CI: 0.37 to 90.37) among persons less than 20 years of age (6 exposed *v.s.* 2 not exposed) and was 0.73 (95% CI: 0.06 to 6.70) among adults (2 exposed *v.s.* 3 not exposed).

## Discussion

The incidence rate of narcolepsy recorded in Quebec, in 2009–2010, (1.5 per million-p-y) was very low compared with the rate (13.7 per million p-y) reported in a previous study in Olmsted County, Minnesota, in 1960–1989 [20]. In Europe, the incidence rates reported in 6 countries in 2000–2010, ranged from a minimum of 1.9 to a maximum of 14.2 per million p-y [21]. In our study, very strict diagnostic criteria were used to define narcolepsy cases and a majority of patients with suspected diagnosis were referred to a single sleep laboratory serving as a reference centre for the province. At the end of a very intense recruitment process, only 3.8% of the total cases investigated were retained. In a retrospective survey in the United-Kingdom, the median delay between the onset of first symptoms and the confirmation of diagnosis was 10.5 years, but this was much shorter in recent years and for cases with cataplexy [22]. In Quebec, the accessibility of primary care services is not great for patients with mild conditions but this should not be the case for young persons with severe symptoms affecting their performance at school or at work. The incidence of narcolepsy in a given population may be influenced by genetic factors [1], [2]. This is not a likely explanation for the low incidence rate reported in our study. Actually, the prevalence of the HLA-DR2 allele, a close marker to the HLA DQB1*0602 predisposing trait [23] has been estimated to 22% in the Quebec population [24], similar to the 21% reported in Caucasian Americans [25], [26].

In our study, the date of onset of EDS was selected to define exposure for all main statistical analyses. The interval between disease onset and first medical visit exceeded two months for most of our patients (median 71 days) and this interval was shorter in exposed cases. Using the date of first medical visit instead of the date of first symptoms introduced a misclassification bias and diluted any effect of vaccination. The problem was even worse using the date of confirmation of diagnosis. The date of first symptoms may be less precise than other points of reference and some recall bias cannot be excluded. In Quebec, however, media attention to the possible association between the pandemic vaccine and narcolepsy was minimal up to the end of 2012, as shown in a survey we performed recently (data not shown).

There was no obvious seasonal pattern in the incidence of narcolepsy in Quebec during the two-year study period. A first cluster of 9 cases was observed in April−August 2009, concomitantly with the first influenza pandemic wave which occurred in May−June 2009. The circulation of seasonal influenza viruses during the winters 2008–2009 and 2009–2010 was unusually low as compared with previous seasons [27]. The second influenza pandemic wave was observed in October−November 2009, with the peak incidence three weeks before the peak of the mass immunization campaign, generating an association which is difficult to disentangle. A temporal association between seasonal and pandemic influenza has been reported in China [28], [29], but not in a less rigorous study in South-Korea [30]. In the second cluster observed in Quebec in the period of October 2009 to March 2010, 7 of the 11 cases were patients who developed symptoms less than 16 weeks after pandemic vaccine administration. These seven cases rapidly developed narcolepsy, with the presence of cataplexy (6/7). Persons less than 20 years of age were over-represented in this sample (5/7). In our study, no information was retrospectively collected on influenza-like symptoms and medical visits for acute infection, and this is a limitation. In a context of wait times in vaccination clinics during the mass campaign, it is reasonable to assume that people who developed an influenza–like illness did not rush to get a vaccine as soon as they recovered. Also, people who received one dose of adjuvanted pandemic vaccine were rapidly protected [31], [32]. It is thus very unlikely that the rate of pandemic influenza infection would have been much higher in the vaccinated portion than in the unvaccinated portion of the population. Also, the 2009–2010 seasonal influenza vaccination campaign in Quebec was postponed and started late in January 2010 with low uptake [15] and this cannot explain the second cluster. In a recent study in Finland [33], no serologic evidence of a contribution of the A/H1N1 (2009) virus infection in the epidemic of childhood narcolepsy could be found. In this study, antibody reactivity to a NS1 polymorphism specific of wild type A/H1N1 (2009) but not present in the vaccine was used to test this hypothesis.

*A priori*, a cohort approach was selected for the base-case analysis as in a previous study on Guillain-Barré Syndrome [17], and the choice of a 16-week post-vaccination risk window, based on series of cases reported in Canada and in Ireland [11], [14]. This is the most natural and statistically powerful approach as risk estimates are based on all persons in the study population observed during the whole study period. Two other methods were used: the SCCS method as described by Whitaker and colleagues [18], which allows for the control of all permanent characteristics of patients, and a traditional matched case-control analysis nested in a cohort, which allows for the control of any seasonal or other cyclic effect [19]. An excess post-vaccination risk was measured in almost all analyses although its magnitude and statistical significance were influenced both by the choice of the risk window and of the reference period.

Our results showing an increased narcolepsy risk in vaccinated individuals less than 20 years of age are congruent with those of studies on a similar AS03 adjuvanted pandemic vaccine in Europe although the magnitude of the reported excess risk was much higher in Norway (90 per million) [34], Finland (63 per million) [9], in Sweden (36 per million) [35], in Ireland (53 per million) [11], and in England (18 per million) [13]. The origin of this difference is not known. Differences in the production process and composition of the two vaccines are a possible hypothesis [36]–[38]. Underascertainment of narcolepsy cases in our study could also bias to some extend attributable risk estimates.

## Conclusions

We can cautiously conclude that our results are consistent with a risk of narcolepsy of small magnitude (approximately one case per million dose) following administration of the adjuvanted (AS03) A/H1N1 pandemic vaccine manufactured in Quebec, and that this risk occurred preferentially in people under 20 years of age with a short latency (≤16 weeks between vaccine administration and disease onset) and with the presence of cataplexy. However, given the small numbers, the impossibility to completely exclude a confounding effect of A/H1N1 (2009) virus infection and/or a reporting bias, our results are to be considered as weak evidence of a causal relationship. We can, however, reasonably exclude the existence of an excess risk of much higher magnitude as reported in some European countries in which Pandemrix had been used. Further studies comparing influenza and autoimmune responses to Pandemrix versus Arepanrix will be needed to shed light on these differences.

## Supporting Information

Table S1 **Standard diagnostic tests performed at HSCM Sleep Disorders Center.**
(DOCX)

Table S2 **List of 24 narcolepsy cases included in final analysis, province of Quebec, 2009–2010.**
(DOCX)

Table S3 **Risk of narcolepsy associated with A/H1N1 (2009) vaccination using the cohort method according to observation period and post-vaccination risk period in persons less than 20 years of age.**
(DOCX)

Table S4 **Risk of narcolepsy associated with A/H1N1 (2009) vaccination using the cohort method according to observation period and post-vaccination risk period in persons ≥20 years of age.**
(DOCX)

Table S5 **Sensitivity analysis in cohort approach: Risk of narcolepsy associated with A/H1N1 vaccination using date of onset of narcolepsy, according to observation period and post-vaccination risk period, and including one case (#009) with date of onset of sleep disorders in 2008 but onset of cataplexy in 2009 which is now considered as the date of disease onset.**
(DOCX)

Table S6 **Sensitivity analysis in cohort approach: Risk of narcolepsy associated with A/H1N1 vaccination using date of onset of narcolepsy, according to observation period and post-vaccination risk period, and including one case (#020) with uncertain date of onset which is now considered as exposed.**
(DOCX)

Table S7 **Sensitivity analysis in cohort approach: Risk of narcolepsy associated with A/H1N1 vaccination using date of onset of cataplexy, according to observation period and post-vaccination risk period, and excluding a case (#011) with uncertain date of onset of cataplexy.**
(DOCX)

Table S8 **Sensitivity analysis in cohort approach: Risk of narcolepsy associated with A/H1N1 vaccination using date of first medical visit, according to observation period and post-vaccination risk period.**
(DOCX)

Table S9 **Risk of narcolepsy using the cohort method according to vaccination status and risk periods defined around the date of vaccination (for non-vaccinated persons a pseudo date was assigned: the median date of vaccination among vaccinated persons of same age and gender).**
(DOCX)
