# Supplementary material for: Risk of Narcolepsy Associated with Inactivated Adjuvanted (AS03) A/H1N1 (2009) Pandemic Influenza Vaccine in Quebec
Source: PLoS One. 2014 Sep 29;9(9):e108489. doi: 10.1371/journal.pone.0108489 (PMC4180737; doi:10.1371/journal.pone.0108489)
Supplement: Table S1 — Standard diagnostic tests performed at HSCM Sleep Disorders Center. (DOCX) [file pone.0108489.s001.docx]

**Table S1: Standard diagnostic tests performed at HSCM Sleep Disorders Center.**

**Polysomnography**

Polysomnographic recordings were conducted overnight on a 32-channel Grass polygraph (sensitivity at 7µV, bandpass at 0.3-100 Hz). EEG recordings and electrode placement were performed according the international 10-20 system. Sleep was recorded and scored according to the standard method (Iber et al., 2007). Respiration was monitored using a nasal canula and a thoracic strain gauge and oxygen saturation was recorded with a finger pulse oxymeter. The Multiple Sleep Latency Test (MSLT), consisting of four or five opportunities to nap, was administered at 10:00, 12:00, 14:00, 16:00, and 18:00 (Carskadon et al., 1986). Sleep latency was defined as the time from lights out to the first epoch of any sleep stage. The test continued for 15 minutes after the first epoch of sleep to assess the occurrence of sleep onset REM period (SOREMP). If sleep did not occur, the test was stopped after 20 min and a sleep latency of 20 minutes was recorded. The mean sleep latency (arithmetic mean of all sleep latencies) and number of SOREMPs were noted. Participants were instructed not to take their psychostimulant or anti-cataplectic medications for two weeks prior to the MSLT. They were not allowed to drink alcohol or beverages containing caffeine, nor were they allowed to sleep between the tests. Smoking has to be stopped at least 30 minutes prior to each nap opportunity (for complete guidelines see Littner et al., 2005).

**Blood sampling**

Samples were sent to Stanford University Center for Narcolepsy, Palo Alto, USA, as tubes of fresh blood. Nucleated cells were separated and DNA extracted for DQB1*06:02 typing (Hallmayer et al., 2009). The presence or absence of DQB1*0602 was determined using DQB1 exon 2 sequence-specific primers. These primers amplify DQB1*0602 and a few exceptionally rare DQB1*06 alleles (allele frequency <0.5%) as a 218-bp PCR product. The assay includes internal positive controls for DNA quality and loading, and both positive and negative controls for DQB1*0602 specificity. Thirty-five cycles at 95°C for 30 sec, 63°C for 30 sec, 72°C for 1 min are used.

**Lumbar puncture and CSF hypocretin-1 assessments**

The lumbar punctures were performed by a neurologist and served to quantify the hypocretin-1 levels in the CSF. It was performed by inserting the needle in the L4-L5 interspace at the level of the iliac crest and drawing off 8 ml of fluid. Cerebrospinal fluid was frozen (at –80°C) immediately upon tapping and shipped frozen in 4 batches to Stanford University Center for Narcolepsy, Palo Alto, USA, where the measurements were performed. CSF hypocretin-1 was measured using a previously published radioimmunoassay (Mignot et al., 2002). The modification is the substitution of the standard antibody with an in house developed antibody with 10x higher affinity. With this modification we can measure hcrt-1 very precisely in 50 µL of crude CSF (detection limit < 40 pg/ml). The cut-off value for pathologically low hcrt-1 is 110 pg/mL based on Receiving Operating Characteristic (ROC) curve analysis versus a clinical gold standard for narcolepsy/cataplexy. A standard CSF sample was used to correct for inter-assay variation. All results were verified in two different assays.
