# Supplementary material for: Risk of Narcolepsy Associated with Inactivated Adjuvanted (AS03) A/H1N1 (2009) Pandemic Influenza Vaccine in Quebec
Source: PLoS One. 2014 Sep 29;9(9):e108489. doi: 10.1371/journal.pone.0108489 (PMC4180737; doi:10.1371/journal.pone.0108489)
Supplement: Table S2 — List of 24 narcolepsy cases included in final analysis, province of Quebec, 2009–2010. (DOCX) [file pone.0108489.s002.docx]

Table S2: List of 24 narcolepsy cases included in final analysis, province of Quebec, 2009-2010

| **Patients #** | **Age at disease onset (years)** | **Date of onset of narcolepsy (sleep disorder)** | **Date of onset of cataplexy** | **Date first medical visit** | **Date of clinical diagnosis of narcolepsy +/- cataplexy** | **Date of PSG+MSLT** | **Brighton**  **classification** | **Date of vaccination** | **Health region** |
| --- | --- | --- | --- | --- | --- | --- | --- | --- | --- |
| 001 | 9 | Avr-09 | Jun-09 | 04-Jun-09 | 28-Aug-09 | 13-Oct-09 | 2 | - | 06-Montréal |
| 002 | 10 | 25-Dec-09 | Jan-10 | 28-Jan-10 | 14-May-10 | 29-Apr-10 | 1 | 02-Dec-09 | 16-Montérégie |
| 003 | 50 | Mars-10 | Mar-10 | 16-Apr-10 | 16-Apr-10 | 02-Jun-10 | 2 | 01-Dec-09 | 16-Montérégie |
| 004 | 13 | Janv-10 | - | 07-Apr-10 | 19-May-11 | 18-May-11 | 3 | 25-Nov-09 | 15-Laurentide |
| 005 | 40 | 30-Nov-09 | 25-Dec-09 | 18-Jan-10 | 20-Jul-10 | 21-Jul-10 | 2 | 09-Nov-09 | 08-Abitibi |
| 006 | 29 | Oct-09 | - | 2-Dec-09 | 31-May-11 | 30-May-11 | 3 | - | 15-Laurentide |
| 007 | 17 | 25-Dec-09 | 25-Dec-09 | 28-Apr-10 | 28-Apr-10 | 25-May-10 | 2 | 19-Dec-09 | 16-Montérégie |
| 008 | 6 | 08-Mars-10 | 05-Apr-10 | 14-Apr-10 | 24-Sep-10 | 22-Nov-10 | 2 | 05-Dec-09 | 06-Montréal |
| 009 | 8 | Sept-08 (?) | 11-Aug-09 | 04-Oct-08 | 23-Feb-09 | 11-Aug-09 | 2 | 12-Nov-09 | 09-Basse-Côte-Nord |
| 010 | 18 | Aug-09 | - | 21-Oct-09 | 26-Aug-10 | 25-Aug-10 | 3 | 30-Nov-09 | 14-Lanaudière |
| 011 | 41 | Aug-09 | Unknown | 18-Aug-09 | 05-Oct-10 | 04-Oct-10 | 2 | 03-Dec-09 | 15-Laurentide |
| 012 | 40 | May-09 | - | 25-May-09 | 28-Jul-10 | 01-Nov-09 | 3 | 12-Nov-09 | 15-Laurentide |
| 013 | 15 | Nov/Dec-09 | Nov/Dec-09 | 25-May-10 | 06-Jul-10 | 05-Jul-10 | 2 | - | 13-Laval |
| 014 | 13 | Feb-10 | Spring-10 | 12-Apr-10 | 25-May-11 | 24-Maii-11 | 1 | 16-Nov-09 | 16-Montérégie |
| 015 | 55 | Jan-10 | Mar-10 | 30-Mar-10 | 17-Jan-11 | 30-Mar-11 | 2 | - | 14-Lanaudière |
| 016 | 32 | May-09 | - | 16-Mar--10 | 21-Dec-10 | 20-Dec-10 | 3 | - | 06-Montréal |
| 017 | 32 | Jun-10 | Jun-10 | 23-Jun-11 | 03-Aug-11 | 02-Aug-11 | 2 | - | 06-Montreal |
| 018 | 29 | 01-Jun-09 | - | 25-Jun-09 | 14-Sep-11 | 08-Nov-11 | 3 | 02-Dec-09 | 04-Mauricie |
| 019 | 10 | Jun-09 | Jun-09 | 04-Sep-09 | 23-Nov-11 | 22-Nov-11 | 1 | 05-Dec-09 | 04-Mauricie |
| 020 | 17 | Oct/Nov-2009 | - | 06-dec-10 | 05-Jan-11 | 04-Jan-11 | 3 | 15-Nov-09 | 06-Montréal |
| 021 | 17 | Jun-09 | Jun-09 | 23-Aug-11 | 03-Nov-11 | 05-Dec-11 | 1 | - | 15-Laurentide |
| 022 | 26 | Jul-10 | Jul-10 | 06-Sep-11 | 31-Dec-11 | 30-Dec-11 | 2 | - | 07-Outaouais |
| 023 | 32 | Jul-09 | 01-Fev-10 | 09-Feb-10 | 04-Jul-10 | 03-Jul-10 | 2 | 04-Dec-09 | 16-Montérégie |
| 024 | 6 | Sep-10 | - | 10-Dec-10 | 05Sep-12 | 26-Sep-12 | 3 | 20-Nov-09 | 06-Montréal |
