# Supplementary material for: Risk of Narcolepsy Associated with Inactivated Adjuvanted (AS03) A/H1N1 (2009) Pandemic Influenza Vaccine in Quebec
Source: PLoS One. 2014 Sep 29;9(9):e108489. doi: 10.1371/journal.pone.0108489 (PMC4180737; doi:10.1371/journal.pone.0108489)
Supplement: Table S3 — Risk of narcolepsy associated with A/H1N1 (2009) vaccination using the cohort method according to observation period and post-vaccination risk period in persons less than 20 years of age. (DOCX) [file pone.0108489.s003.docx]

Table S3: Risk of narcolepsy associated with A/H1N1 (2009) vaccination using the cohort method according to observation period and post-vaccination risk period in persons less than 20 years of age

|  |  | **No cases** | | | **Rate/100 000 Person-years** | | | **Attributable cases/ million doses** | **Crude risk ratio (95% CI)** | | | |
| --- | --- | --- | --- | --- | --- | --- | --- | --- | --- | --- | --- | --- |
|  |  | *E+* | *E-* | *Total* | *E+* | *E-* | *Total* |  | *RR* | *IC_inf_* | *IC_sup_* | *P-value* |
| **Observation period** | **Risk period from date of vaccination to:** |  |  |  |  |  |  |  |  |  |  |  |
| **January 01, 2009 - December 31, 2010** | End study period : Dec 31st, 2010 | 6 | 6 | 12 | 0.483 | 0.280 | 0.354 | 2.270 | 1.73 | 0.46 | 6.46 | 0.503 |
|  | 365 days (1 year) post-vaccination | 6 | 6 | 12 | 0.540 | 0.263 | 0.354 | 2.767 | 2.05 | 0.55 | 7.67 | 0.333 |
|  | 168 days (24 weeks) post-vaccination | 5 | 7 | 12 | 0.978 | 0.243 | 0.354 | 3.380 | 4.02 | 1.00 | 14.71 | 0.049 |
|  | 112 days (16 weeks) post-vaccination* | 5 | 7 | 12 | 1.467 | 0.230 | 0.354 | 3.794 | 6.39 | 1.60 | 23.38 | 0.009 |
|  | 56 days (8 weeks) post-vaccination | 3 | 9 | 12 | 1.761 | 0.280 | 0.354 | 2.270 | 6.29 | 1.10 | 25.22 | 0.039 |
| **May 01, 2009 - March 31, 2010** | End study period : Dec 31^st^, 2010 | 5 | 5 | 10 | 1.231 | 0.435 | 0.643 | 2.907 | 2.83 | 0.65 | 12.28 | 0.184 |
|  | 365 days (1 year) post-vaccination | 5 | 5 | 10 | 1.231 | 0.435 | 0.643 | 2.907 | 2.83 | 0.65 | 12.28 | 0.184 |
|  | 168 days (24 weeks) post-vaccination | 5 | 5 | 10 | 1.231 | 0.435 | 0.643 | 2.907 | 2.83 | 0.65 | 12.28 | 0.184 |
|  | 112 days (16 weeks) post-vaccination | 5 | 5 | 10 | 1.467 | 0.412 | 0.643 | 3.236 | 3.56 | 0.82 | 15.48 | 0.094 |
|  | 56 days (8 weeks) post-vaccination | 3 | 7 | 10 | 1.761 | 0.506 | 0.643 | 1.924 | 3.48 | 0.58 | 15.26 | 0.175 |
| **October 04, 2009 - March 31, 2010** | End study period : Dec 31^st^, 2010 | 5 | 2 | 7 | 1.231 | 0.471 | 0.843 | 2.776 | 2.61 | 0.43 | 27.43 | 0.418 |
|  | 365 days (1 year) post-vaccination | 5 | 2 | 7 | 1.231 | 0.471 | 0.843 | 2.776 | 2.61 | 0.43 | 27.43 | 0.418 |
|  | 168 days (24 weeks) post-vaccination | 5 | 2 | 7 | 1.231 | 0.471 | 0.843 | 2.776 | 2.61 | 0.43 | 27.43 | 0.418 |
|  | 112 days (16 weeks) post-vaccination | 5 | 2 | 7 | 1.467 | 0.408 | 0.843 | 3.247 | 3.59 | 0.59 | 37.75 | 0.213 |
|  | 56 days (8 weeks) post-vaccination | 3 | 4 | 7 | 1.761 | 0.606 | 0.843 | 1.771 | 2.91 | 0.43 | 17.19 | 0.314 |

*E+: Cases with onset after vaccination during risk period; E-: Cases not vaccinated or with onset before vaccination or after end of risk period*

**Base analysis defined a priori*
