# Supplementary material for: Risk of Narcolepsy Associated with Inactivated Adjuvanted (AS03) A/H1N1 (2009) Pandemic Influenza Vaccine in Quebec
Source: PLoS One. 2014 Sep 29;9(9):e108489. doi: 10.1371/journal.pone.0108489 (PMC4180737; doi:10.1371/journal.pone.0108489)
Supplement: Table S4 — Risk of narcolepsy associated with A/H1N1 (2009) vaccination using the cohort method according to observation period and post-vaccination risk period in persons ≥20 years of age. (DOCX) [file pone.0108489.s004.docx]

Table S4: Risk of narcolepsy associated with A/H1N1 (2009) vaccination using the cohort method according to observation period and post-vaccination risk period in persons ≥ 20 years of age

|  |  | **No cases** | | | **Rate/100 000 Person-years** | | | **Attributable cases/ million doses** | **Crude risk ratio (95% CI)** | | | |
| --- | --- | --- | --- | --- | --- | --- | --- | --- | --- | --- | --- | --- |
|  |  | *E+* | *E-* | *Total* | *E+* | *E-* | *Total* |  | *RR* | *IC_inf_* | *IC_sup_* | *P-value* |
| **Observation period** | **Risk period from date of vaccination to:** |  |  |  |  |  |  |  |  |  |  |  |
| **January 01, 2009 - December 31, 2010** | End study period : Dec 31st, 2010 | 2 | 9 | 11 | 0.055 | 0.106 | 0.090 | -0.560 | 0.52 | 0.05 | 2.51 | 0.626 |
|  | 365 days (1 year) post-vaccination | 2 | 9 | 11 | 0.060 | 0.102 | 0.090 | -0.411 | 0.60 | 0.06 | 2.87 | 0.779 |
|  | 168 days (24 weeks) post-vaccination | 2 | 9 | 11 | 0.131 | 0.085 | 0.090 | 0.215 | 1.55 | 0.16 | 7.51 | 0.817 |
|  | 112 days (16 weeks) post-vaccination* | 2 | 9 | 11 | 0.197 | 0.081 | 0.090 | 0.357 | 2.44 | 0.26 | 11.80 | 0.465 |
|  | 56 days (8 weeks) post-vaccination | 1 | 10 | 11 | 0.197 | 0.086 | 0.090 | 0.171 | 2.30 | 0.05 | 16.15 | 0.748 |
| **May 01, 2009 - March 31, 2010** | End study period : Dec 31^st^, 2010 | 2 | 7 | 9 | 0.173 | 0.158 | 0.161 | 0.052 | 1.09 | 0.11 | 5.75 | 1.000 |
|  | 365 days (1 year) post-vaccination | 2 | 7 | 9 | 0.173 | 0.158 | 0.161 | 0.052 | 1.09 | 0.11 | 5.75 | 1.000 |
|  | 168 days (24 weeks) post-vaccination | 2 | 7 | 9 | 0.173 | 0.158 | 0.161 | 0.052 | 1.09 | 0.11 | 5.75 | 1.000 |
|  | 112 days (16 weeks) post-vaccination | 2 | 7 | 9 | 0.197 | 0.153 | 0.161 | 0.134 | 1.29 | 0.13 | 6.75 | 1.000 |
|  | 56 days (8 weeks) post-vaccination | 1 | 8 | 9 | 0.197 | 0.158 | 0.161 | 0.060 | 1.25 | 0.03 | 9.33 | 1.000 |
| **October 04, 2009 - March 31, 2010** | End study period : Dec 31^st^, 2010 | 2 | 1 | 3 | 0.173 | 0.055 | 0.101 | 0.413 | 3.16 | 0.16 | 186.40 | 0.668 |
|  | 365 days (1 year) post-vaccination | 2 | 1 | 3 | 0.173 | 0.055 | 0.101 | 0.413 | 3.16 | 0.16 | 186.40 | 0.668 |
|  | 168 days (24 weeks) post-vaccination | 2 | 1 | 3 | 0.173 | 0.055 | 0.101 | 0.413 | 3.16 | 0.16 | 186.40 | 0.668 |
|  | 112 days (16 weeks) post-vaccination | 2 | 1 | 3 | 0.197 | 0.051 | 0.101 | 0.448 | 3.88 | 0.20 | 228.80 | 0.537 |
|  | 56 days (8 weeks) post-vaccination | 1 | 2 | 3 | 0.197 | 0.081 | 0.101 | 0.178 | 2.44 | 0.04 | 46.86 | 0.857 |

*E+: Cases with onset after vaccination during risk period; E-: Cases not vaccinated or with onset before vaccination or after end of risk period*

**Primary analysis*
