# Supplementary material for: Risk of Narcolepsy Associated with Inactivated Adjuvanted (AS03) A/H1N1 (2009) Pandemic Influenza Vaccine in Quebec
Source: PLoS One. 2014 Sep 29;9(9):e108489. doi: 10.1371/journal.pone.0108489 (PMC4180737; doi:10.1371/journal.pone.0108489)
Supplement: Table S5 — Sensitivity analysis in cohort approach: Risk of narcolepsy associated with A/H1N1 vaccination using date of onset of narcolepsy, according to observation period and post-vaccination risk period, and including one case (#009) with date of onset of sleep disorders in 2008 but onset of cataplexy in 2009 which is now considered as the date of disease onset. (DOCX) [file pone.0108489.s005.docx]

Table S5: Sensitivity analysis in cohort approach: Risk of narcolepsy associated with A/H1N1 vaccination using date of onset of narcolepsy, according to observation period and post-vaccination risk period, and including one case (#009) with date of onset of sleep disorders in 2008 but onset of cataplexy in 2009 which is now considered as the date of disease onset

|  |  | **No cases** | | | **Rate/100 000 person-years** | | | **Attributable cases/ million doses** | **Age- and gender-adjusted risk ratio**  **(95% CI)** | | | |
| --- | --- | --- | --- | --- | --- | --- | --- | --- | --- | --- | --- | --- |
|  |  | *E+* | *E-* | *Total* | *E+* | *E-* | *Total* |  | *RR ajusté* | *IC_inf_* | *IC_sup_* | *P-value* |
| **Observation period** | **Risk period from date of vaccination to:** |  |  |  |  |  |  |  |  |  |  |  |
| **January 01, 2009 - December 31, 2010** | End study period : Dec 31st, 2010 | 8 | 16 | 24 | 0.164 | 0.150 | 0.154 | 0.149 | 0.99 | 0.37 | 2.46 | 1.00 |
|  | 365 days (1 year) post-vaccination | 8 | 16 | 24 | 0.181 | 0.144 | 0.154 | 0.374 | 1.16 | 0.43 | 2.88 | 0.882 |
|  | 168 days (24 weeks) post-vaccination | 7 | 17 | 24 | 0.344 | 0.126 | 0.154 | 1.005 | 2.56 | 0.89 | 6.52 | 0.079 |
|  | 112 days (16 weeks) post-vaccination | 7 | 17 | 24 | 0.516 | 0.120 | 0.154 | 1.216 | 4.05 | 1.42 | 10.30 | 0.009 |
|  | 56 days (8 weeks) post-vaccination | 4 | 20 | 24 | 0.590 | 0.134 | 0.154 | 0.698 | 4.13 | 1.03 | 12.35 | 0.046 |
| **May 01, 2009 - March 31, 2010** | End study period : Dec 31^st^, 2010 | 7 | 13 | 20 | 0.448 | 0.233 | 0.280 | 0.759 | 1.75 | 0.59 | 4.73 | 0.344 |
|  | 365 days (1 year) post-vaccination | 7 | 13 | 20 | 0.448 | 0.233 | 0.280 | 0.759 | 1.75 | 0.59 | 4.73 | 0.344 |
|  | 168 days (24 weeks) post-vaccination | 7 | 13 | 20 | 0.448 | 0.233 | 0.280 | 0.759 | 1.75 | 0.59 | 4.73 | 0.344 |
|  | 112 days (16 weeks) post-vaccination | 7 | 13 | 20 | 0.516 | 0.225 | 0.280 | 0.894 | 2.13 | 0.72 | 5.78 | 0.179 |
|  | 56 days (8 weeks) post-vaccination | 4 | 16 | 20 | 0.590 | 0.248 | 0.280 | 0.525 | 2.23 | 0.54 | 6.93 | 0.272 |
| **October 04, 2009 - March 31, 2010** | End study period : Dec 31^st^, 2010 | 7 | 3 | 10 | 0.448 | 0.133 | 0.262 | 1.112 | 2.73 | 0.62 | 16.45 | 0.234 |
|  | 365 days (1 year) post-vaccination | 7 | 3 | 10 | 0.448 | 0.133 | 0.262 | 1.112 | 2.73 | 0.62 | 16.45 | 0.234 |
|  | 168 days (24 weeks) post-vaccination | 7 | 3 | 10 | 0.448 | 0.133 | 0.262 | 1.112 | 2.73 | 0.62 | 16.45 | 0.234 |
|  | 112 days (16 weeks) post-vaccination | 7 | 3 | 10 | 0.516 | 0.122 | 0.262 | 1.209 | 3.63 | 0.82 | 21.82 | 0.098 |
|  | 56 days (8 weeks) post-vaccination | 4 | 6 | 10 | 0.590 | 0.191 | 0.262 | 0.611 | 2.73 | 0.57 | 11.55 | 0.229 |

*E+: Cases with onset after vaccination during risk period; E-: Cases not vaccinated or with onset before vaccination or after end of risk period*
