# Supplementary material for: Risk of Narcolepsy Associated with Inactivated Adjuvanted (AS03) A/H1N1 (2009) Pandemic Influenza Vaccine in Quebec
Source: PLoS One. 2014 Sep 29;9(9):e108489. doi: 10.1371/journal.pone.0108489 (PMC4180737; doi:10.1371/journal.pone.0108489)
Supplement: Table S6 — Sensitivity analysis in cohort approach: Risk of narcolepsy associated with A/H1N1 vaccination using date of onset of narcolepsy, according to observation period and post-vaccination risk period, and including one case (#020) with uncertain date of onset which is now considered as exposed. (DOCX) [file pone.0108489.s006.docx]

Table S6: Sensitivity analysis in cohort approach: Risk of narcolepsy associated with A/H1N1 vaccination using date of onset of narcolepsy, according to observation period and post-vaccination risk period, and including one case (#020) with uncertain date of onset which is now considered as exposed

|  |  | **No cases** | | | **Rate/100 000 person-years** | | | **Attributable cases/ million doses** | **Age- and gender-adjusted risk ratio (95% CI)** | | | |
| --- | --- | --- | --- | --- | --- | --- | --- | --- | --- | --- | --- | --- |
|  |  | *E+* | *E-* | *Total* | *E+* | *E-* | *Total* |  | *RR ajusté* | *IC_inf_* | *IC_sup_* | *P-value* |
| **Observation period** | **Risk period from date of vaccination to:** |  |  |  |  |  |  |  |  |  |  |  |
| **January 01, 2009 - December 31, 2010** | End study period : Dec 31st, 2010 | 9 | 14 | 23 | 0.184 | 0.131 | 0.148 | 0.583 | 1.28 | 0.49 | 3.19 | 0.70 |
|  | 365 days (1 year) post-vaccination | 9 | 14 | 23 | 0.204 | 0.126 | 0.148 | 0.779 | 1.50 | 0.57 | 3.73 | 0.460 |
|  | 168 days (24 weeks) post-vaccination | 8 | 15 | 23 | 0.393 | 0.111 | 0.148 | 1.299 | 3.33 | 1.22 | 8.39 | 0.019 |
|  | 112 days (16 weeks) post-vaccination | 8 | 15 | 23 | 0.590 | 0.106 | 0.148 | 1.485 | 5.27 | 1.93 | 13.27 | 0.001 |
|  | 56 days (8 weeks) post-vaccination | 5 | 18 | 23 | 0.737 | 0.121 | 0.148 | 0.945 | 5.76 | 1.67 | 16.13 | 0.007 |
| **May 01, 2009 - March 31, 2010** | End study period : Dec 31^st^, 2010 | 8 | 11 | 19 | 0.512 | 0.197 | 0.266 | 1.112 | 2.38 | 0.83 | 6.53 | 0.112 |
|  | 365 days (1 year) post-vaccination | 8 | 11 | 19 | 0.512 | 0.197 | 0.266 | 1.112 | 2.38 | 0.83 | 6.53 | 0.112 |
|  | 168 days (24 weeks) post-vaccination | 8 | 11 | 19 | 0.512 | 0.197 | 0.266 | 1.112 | 2.38 | 0.83 | 6.53 | 0.112 |
|  | 112 days (16 weeks) post-vaccination | 8 | 11 | 19 | 0.590 | 0.190 | 0.266 | 1.226 | 2.90 | 1.01 | 7.95 | 0.047 |
|  | 56 days (8 weeks) post-vaccination | 5 | 14 | 19 | 0.738 | 0.217 | 0.266 | 0.798 | 3.20 | 0.90 | 9.44 | 0.071 |
| **October 04, 2009 - March 31, 2010** | End study period : Dec 31^st^, 2010 | 8 | 2 | 10 | 0.512 | 0.089 | 0.262 | 1.495 | 4.69 | 0.93 | 45.65 | 0.065 |
|  | 365 days (1 year) post-vaccination | 8 | 2 | 10 | 0.512 | 0.089 | 0.262 | 1.495 | 4.69 | 0.93 | 45.65 | 0.065 |
|  | 168 days (24 weeks) post-vaccination | 8 | 2 | 10 | 0.512 | 0.089 | 0.262 | 1.495 | 4.69 | 0.93 | 45.65 | 0.065 |
|  | 112 days (16 weeks) post-vaccination | 8 | 2 | 10 | 0.590 | 0.081 | 0.262 | 1.560 | 6.23 | 1.24 | 60.44 | 0.021 |
|  | 56 days (8 weeks) post-vaccination | 5 | 5 | 10 | 0.738 | 0.159 | 0.262 | 0.886 | 4.10 | 0.94 | 17.88 | 0.061 |

*E+: Cases with onset after vaccination during risk period; E-: Cases not vaccinated or with onset before vaccination or after end of risk period*
