# Supplementary material for: Risk of Narcolepsy Associated with Inactivated Adjuvanted (AS03) A/H1N1 (2009) Pandemic Influenza Vaccine in Quebec
Source: PLoS One. 2014 Sep 29;9(9):e108489. doi: 10.1371/journal.pone.0108489 (PMC4180737; doi:10.1371/journal.pone.0108489)
Supplement: Table S7 — Sensitivity analysis in cohort approach: Risk of narcolepsy associated with A/H1N1 vaccination using date of onset of cataplexy, according to observation period and post-vaccination risk period, and excluding a case (#011) with uncertain date of onset of cataplexy. (DOCX) [file pone.0108489.s007.docx]

Table S7: Sensitivity analysis in cohort approach: Risk of narcolepsy associated with A/H1N1 vaccination using date of onset of cataplexy, according to observation period and post-vaccination risk period, and excluding a case (#011) with uncertain date of onset of cataplexy

|  |  | **No cases** | | | **Rate/100 000 person-years** | | | **Attributable cases/ million doses** | **Age- and gender-adjusted risk ratio (95% CI)** | | | |
| --- | --- | --- | --- | --- | --- | --- | --- | --- | --- | --- | --- | --- |
|  |  | *E+* | *E-* | *Total* | *E+* | *E-* | *Total* |  | *RR ajusté* | *IC_inf_* | *IC_sup_* | *P-value* |
| **Observation period** | **Risk period from date of vaccination to:** |  |  |  |  |  |  |  |  |  |  |  |
| **January 01, 2009 - December 31, 2010** | End study period : Dec 31st, 2010 | 7 | 8 | 15 | 0.143 | 0.075 | 0.096 | 0.753 | 1.71 | 0.52 | 5.42 | 0.44 |
|  | 365 days (1 year) post-vaccination | 7 | 8 | 15 | 0.158 | 0.072 | 0.096 | 0.865 | 2.00 | 0.62 | 6.34 | 0.279 |
|  | 168 days (24 weeks) post-vaccination | 7 | 8 | 15 | 0.344 | 0.059 | 0.096 | 1.311 | 5.39 | 1.66 | 17.04 | 0.005 |
|  | 112 days (16 weeks) post-vaccination | 5 | 10 | 15 | 0.369 | 0.070 | 0.096 | 0.915 | 4.86 | 1.30 | 15.64 | 0.019 |
|  | 56 days (8 weeks) post-vaccination | 3 | 12 | 15 | 0.442 | 0.081 | 0.096 | 0.555 | 5.10 | 0.92 | 18.95 | 0.061 |
| **May 01, 2009 - March 31, 2010** | End study period : Dec 31^st^, 2010 | 5 | 6 | 11 | 0.320 | 0.108 | 0.154 | 0.750 | 2.67 | 0.81 | 8.79 | 0.106 |
|  | 365 days (1 year) post-vaccination | 5 | 6 | 11 | 0.320 | 0.108 | 0.154 | 0.750 | 2.67 | 0.81 | 8.79 | 0.106 |
|  | 168 days (24 weeks) post-vaccination | 5 | 6 | 11 | 0.320 | 0.108 | 0.154 | 0.750 | 2.67 | 0.81 | 8.79 | 0.106 |
|  | 112 days (16 weeks) post-vaccination | 5 | 6 | 11 | 0.369 | 0.104 | 0.154 | 0.812 | 3.26 | 0.78 | 12.85 | 0.109 |
|  | 56 days (8 weeks) post-vaccination | 3 | 8 | 11 | 0.443 | 0.124 | 0.154 | 0.489 | 3.30 | 0.56 | 13.80 | 0.188 |
| **October 04, 2009 - March 31, 2010** | End study period : Dec 31^st^, 2010 | 5 | 2 | 7 | 0.320 | 0.089 | 0.183 | 0.817 | 3.38 | 0.54 | 35.83 | 0.251 |
|  | 365 days (1 year) post-vaccination | 5 | 2 | 7 | 0.320 | 0.089 | 0.183 | 0.817 | 3.38 | 0.54 | 35.83 | 0.251 |
|  | 168 days (24 weeks) post-vaccination | 5 | 2 | 7 | 0.320 | 0.089 | 0.183 | 0.817 | 3.38 | 0.54 | 35.83 | 0.251 |
|  | 112 days (16 weeks) post-vaccination | 5 | 2 | 7 | 0.369 | 0.081 | 0.183 | 0.881 | 4.32 | 0.70 | 45.69 | 0.137 |
|  | 56 days (8 weeks) post-vaccination | 3 | 4 | 7 | 0.443 | 0.128 | 0.183 | 0.483 | 3.33 | 0.49 | 19.79 | 0.244 |

*E+: Cases with onset after vaccination during risk period; E-: Cases not vaccinated or with onset before vaccination or after end of risk period*
