# Supplementary material for: Risk of Narcolepsy Associated with Inactivated Adjuvanted (AS03) A/H1N1 (2009) Pandemic Influenza Vaccine in Quebec
Source: PLoS One. 2014 Sep 29;9(9):e108489. doi: 10.1371/journal.pone.0108489 (PMC4180737; doi:10.1371/journal.pone.0108489)
Supplement: Table S8 — Sensitivity analysis in cohort approach: Risk of narcolepsy associated with A/H1N1 vaccination using date of first medical visit, according to observation period and post-vaccination risk period. (DOCX) [file pone.0108489.s008.docx]

Table S8: Sensitivity analysis in cohort approach: Risk of narcolepsy associated with A/H1N1 vaccination using date of first medical visit, according to observation period and post-vaccination risk period

|  |  | **No cases** | | | **Rate/100 000 person-years** | | | **Attributable cases/ million doses** | **Age- and gender-adjusted risk ratio (95% CI)** | | | |
| --- | --- | --- | --- | --- | --- | --- | --- | --- | --- | --- | --- | --- |
|  |  | *E+* | *E-* | *Total* | *E+* | *E-* | *Total* |  | *RR ajusté* | *IC_inf_* | *IC_sup_* | *P-value* |
| **Observation period** | **Risk period from date of vaccination to:** |  |  |  |  |  |  |  |  |  |  |  |
| **January 01, 2009 - December 31, 2010** | End study period : Dec 31st, 2010 | 10 | 10 | 20 | 0.204 | 0.094 | 0.129 | 1.224 | 1.99 | 0.74 | 5.35 | 0.19 |
|  | 365 days (1 year) post-vaccination | 8 | 12 | 20 | 0.181 | 0.108 | 0.129 | 0.733 | 1.55 | 0.55 | 4.13 | 0.465 |
|  | 168 days (24 weeks) post-vaccination | 8 | 12 | 20 | 0.393 | 0.089 | 0.129 | 1.401 | 4.15 | 1.47 | 11.07 | 0.007 |
|  | 112 days (16 weeks) post-vaccination | 3 | 17 | 20 | 0.221 | 0.120 | 0.129 | 0.311 | 1.73 | 0.32 | 5.99 | 0.562 |
|  | 56 days (8 weeks) post-vaccination | 0 | 20 | 20 | 0.000 | 0.134 | 0.129 | 0.000 | 0.00 |  |  |  |
| **May 01, 2009 - March 31, 2010** | End study period : Dec 31^st^, 2010 | 3 | 9 | 12 | 0.192 | 0.161 | 0.168 | 0.108 | 1.18 | 0.20 | 4.75 | 1.000 |
|  | 365 days (1 year) post-vaccination | 3 | 9 | 12 | 0.192 | 0.161 | 0.168 | 0.108 | 1.18 | 0.20 | 4.75 | 1.000 |
|  | 168 days (24 weeks) post-vaccination | 3 | 9 | 12 | 0.192 | 0.161 | 0.168 | 0.108 | 1.18 | 0.20 | 4.75 | 1.000 |
|  | 112 days (16 weeks) post-vaccination | 3 | 9 | 12 | 0.221 | 0.156 | 0.168 | 0.201 | 1.42 | 0.24 | 5.69 | 0.818 |
|  | 56 days (8 weeks) post-vaccination | 0 | 12 | 12 | 0.000 | 0.186 | 0.168 | 0.000 | 0.00 |  |  |  |
| **October 04, 2009 - March 31, 2010** | End study period : Dec 31^st^, 2010 | 3 | 4 | 7 | 0.192 | 0.178 | 0.183 | 0.051 | 1.03 | 0.15 | 6.15 | 1.000 |
|  | 365 days (1 year) post-vaccination | 3 | 4 | 7 | 0.192 | 0.178 | 0.183 | 0.051 | 1.03 | 0.15 | 6.15 | 1.000 |
|  | 168 days (24 weeks) post-vaccination | 3 | 4 | 7 | 0.192 | 0.178 | 0.183 | 0.051 | 1.03 | 0.15 | 6.15 | 1.000 |
|  | 112 days (16 weeks) post-vaccination | 3 | 4 | 7 | 0.221 | 0.163 | 0.183 | 0.180 | 1.31 | 0.19 | 7.81 | 1.000 |
|  | 56 days (8 weeks) post-vaccination | 0 | 7 | 7 | 0.000 | 0.223 | 0.183 | 0.000 | 0.00 |  |  |  |

*E+: Cases with onset after vaccination during risk period; E-: Cases not vaccinated or with onset before vaccination or after end of risk period*
