# Supplementary material for: Risk of Narcolepsy Associated with Inactivated Adjuvanted (AS03) A/H1N1 (2009) Pandemic Influenza Vaccine in Quebec
Source: PLoS One. 2014 Sep 29;9(9):e108489. doi: 10.1371/journal.pone.0108489 (PMC4180737; doi:10.1371/journal.pone.0108489)
Supplement: Table S9 — Risk of narcolepsy using the cohort method according to vaccination status and risk periods defined around the date of vaccination (for non-vaccinated persons a pseudo date was assigned: the median date of vaccination among vaccinated persons of same age and gender). (DOCX) [file pone.0108489.s009.docx]

Table S9: Risk of narcolepsy using the cohort method according to vaccination status and risk periods defined around the date of vaccination (for non-vaccinated persons a pseudo date was assigned: the median date of vaccination among vaccinated persons of same age and gender)

|  |  | **No cases** | | | **Rate/100 000 Person-years** | | | **Crude risk ratio**  **(95% CI)** | | | |
| --- | --- | --- | --- | --- | --- | --- | --- | --- | --- | --- | --- |
|  |  | ***Post*** | ***Pre*** | *Total* | ***Post*** | ***Pre*** | *Total* | *RR* | *CI_inf_* | *CI_sup_* | *P-value* |
| **Comparison** | **Risk period** |  |  |  |  |  |  |  |  |  |  |
| **Vaccinated persons:** | ±168 days (±24 weeks) | 7 | 4 | 11 | 0.344 | 0.197 | 0.270 | 1.75 | 0.44 | 8.15 | 0.549 |
| **post vs pre-vaccination risk periods** | ±112 days (±16 weeks) | 7 | 3 | 10 | 0.516 | 0.221 | 0.369 | 2.33 | 0.53 | 13.98 | 0.344 |
|  | ±56 days (±8 weeks) | 4 | 1 | 5 | 0.590 | 0.147 | 0.369 | 4.00 | 0.39 | 197.00 | 0.375 |
| **Non-vaccinated persons:** | ±168 days (±24 weeks) | 2 | 2 | 4 | 0.129 | 0.129 | 0.129 | 1.00 | 0.07 | 13.8 | 1.00 |
| **post vs pre-vaccination risk periods** | ±112 days (±16 weeks) | 2 | 1 | 3 | 0.194 | 0.097 | 0.146 | 2.00 | 010 | 118.00 | 1.00 |
|  | ±56 days (±8 weeks) | 2 | 0 | 2 | 0.388 | 0.000 | 0.194 | NA | - | - | - |
|  |  | ***V+*** | ***V-*** | *Total* | **V+** | **V-** | *Total* |  |  |  |  |
| **Post-vaccination period:** | +168 days (+24 weeks) | 7 | 2 | 9 | 0.344 | 0.129 | 0.251 | 2.66 | 0.51 | 26.25 | 0.353 |
| **vaccinated vs non vaccinated persons** | +112 days (+16 weeks) | 7 | 2 | 9 | 0.516 | 0.194 | 0.377 | 2.66 | 0.51 | 26.25 | 0.353 |
|  | +56 days (+8 weeks) | 4 | 2 | 6 | 0.590 | 0.388 | 0.503 | 1.52 | 0.22 | 16.81 | 0.957 |
| **Pre-vaccination period:** | -168 days (-24 weeks) | 4 | 2 | 6 | 0.197 | 0.129 | 0.168 | 1.52 | 0.22 | 16.81 | 0.957 |
| **vaccinated vs non vaccinated persons** | -112 days (-16 weeks) | 3 | 1 | 4 | 0.221 | 0.097 | 0.168 | 2.28 | 0.18 | 119.7 | 0.842 |
|  | -56 days (-8 weeks) | 1 | 0 | 1 | 0.147 | 0.000 | 0.084 | NA | - | - | - |
